# *Candida albicans* enhances iron uptake to maintain fluconazole resistance

**DOI:** 10.1128/iai.00002-25

**Published:** 2025-02-07

**Authors:** Rishabh Sharma, Anubhav Nahar, Sumant Puri

**Affiliations:** 1 Oral Microbiome Research Laboratory, Kornberg School of Dentistry, Temple University 70067 , Philadelphia, Pennsylvania, USA; NIAID, NIH, Washington, DC, USA

**Keywords:** *Candida albicans*, fluconazole, iron, antifungal drug-resistance, β-glucan, ergosterol, oropharyngeal candidiasis, deferasirox, iron chelator

## Abstract

Widespread use of fluconazole has led to the emergence of fluconazole-resistant (FR) *Candid*a spp. causing challenges in clinical treatment. Iron, an essential nutrient, affects the levels of ergosterol (a fluconazole target) in fungal membranes. Our lab-generated FR strain (fluconazole minimum inhibitory concentration [MIC] >125 µg/mL) showed a twofold lower MIC (4.66 µg/mL) for the iron chelator deferasirox (DFX), compared to its patent strain CAI4 (DFX MIC 9.34 µg/mL), suggesting a greater sensitivity to iron chelation. A sublethal dose of DFX (2.33 µg/mL) was able to effectively synergize with 125 µg/mL fluconazole to kill the FR strain. Iron estimation revealed significantly enhanced intracellular iron accumulation in the FR strain compared to CAI4. Expression of iron-uptake genes (*FRP1*, *FRE10*, and *RBT5*) was also significantly upregulated in the FR strain, particularly under high iron. FR strain also showed an increase in the levels of cellular ergosterol, along with an increase in the expression of ergosterol biosynthesis genes (*ERG11* and *ERG9*), compared to CAI4, under both low and high iron. The strain further showed increased β-glucan levels and exposure. Additionally, FR strain showed significantly higher survival in high-iron mice compared to low-iron mice, during fluconazole treatment. Finally, we observed a synergistic fungicidal response between 2.33 µg/mL DFX and 125 µg/mL fluconazole, for FR clinical strains. Overall, this suggests that FR *C. albicans* actively uptakes more iron to maintain cellular conditions needed to support its resistance against fluconazole; and that DFX alone or in conjugation with fluconazole has the potential to overcome fluconazole drug resistance.

## INTRODUCTION

*Candida albicans* is responsible for a wide range of infections, including superficial infections such as oral and vaginal candidiasis (1, 2) as well as more severe invasive infections in immunocompromised individuals (3, 4). Fluconazole, a widely used first-line antifungal agent, has conventionally been effective against *C. albicans* (5). However, the emergence of azole-resistant strains poses a serious challenge to the management of *Candida* infections (5, 6). *C. albicans* has the ability to develop fluconazole resistance through various mechanisms (7–12). Fluconazole inhibits the activity of the cytochrome P-450 enzyme 14-demethylase (ERG11), which is essential for converting lanosterol to ergosterol, a critical component of fungal cell membranes. The most common mechanism of fluconazole resistance is a mutation in the *ERG11* gene, which affects ergosterol synthesis (13). These mutations lead to either the overexpression of the enzyme itself (14) or alterations in its activity that can lower fluconazole’s binding strength, leading to reduced drug effectiveness (15). Drug efflux pumps, such as Cdr1, Cdr2, and Mdr1, also play a crucial role in mediating fluconazole resistance in *C. albicans*, as these are often upregulated in response to fluconazole exposure (8, 9). They actively pump fluconazole out of the cell, contributing to reduced drug accumulation and enhanced resistance. Few studies have shown that fluconazole-resistant (FR) *C. albicans* may also exhibit higher levels of β-glucans compared to susceptible strains (12, 16). β-glucans can act as a physical barrier, reducing the accessibility of fluconazole to its target sites in the cell membrane and limiting its effectiveness (12). Thus, higher glucans can also contribute to reduced drug susceptibility.

Iron is an indispensable micronutrient essential for the pathogenesis and survival of fungal pathogens (17). *C. albicans* employs several mechanisms to obtain iron from its host and is constantly engaged in a contest with the host for control over this vital resource (18–21). Modulation of iron levels alters *C. albicans* dissemination, infection severity, membrane ergosterol levels, and host immune response (largely mediated by iron-induced changes in the levels of immunogenic glucans in the cell wall) (22–25). In the context of antifungal drug resistance, iron availability can influence the efficacy of commonly used antifungal agents (24). The use of iron chelators in combination with fluconazole has been explored previously as a potential synergistic approach to treat infections caused by pathogenic yeast such as *Candida* spp. (26) and *C. neoformans* (27). How changes in iron uptake efficiency may affect membrane ergosterol and wall glucans to maintain fluconazole resistance has not been explored.

This study shows that FR strain of *C. albicans* exhibited increased sensitivity to a Food and Drug Administration (FDA)-approved iron chelator, DFX, when compared to its parent strain. FR strain accumulated significantly higher iron levels to induce high iron-mediated augmented ergosterol levels, as well as higher β-glucan levels and exposure. As both higher glucan exposure and ergosterol biosynthesis facilitate fluconazole resistance, FR strain showed greater survival upon fluconazole treatment in a high iron host. This is the first study to underscore enhanced iron uptake as a mechanism for the maintenance of fluconazole resistance in *C. albicans*.

## RESULTS

### Iron chelation alone or in combination with fluconazole is an effective antifungal strategy against FR *C. albicans*

**Fig 1:**
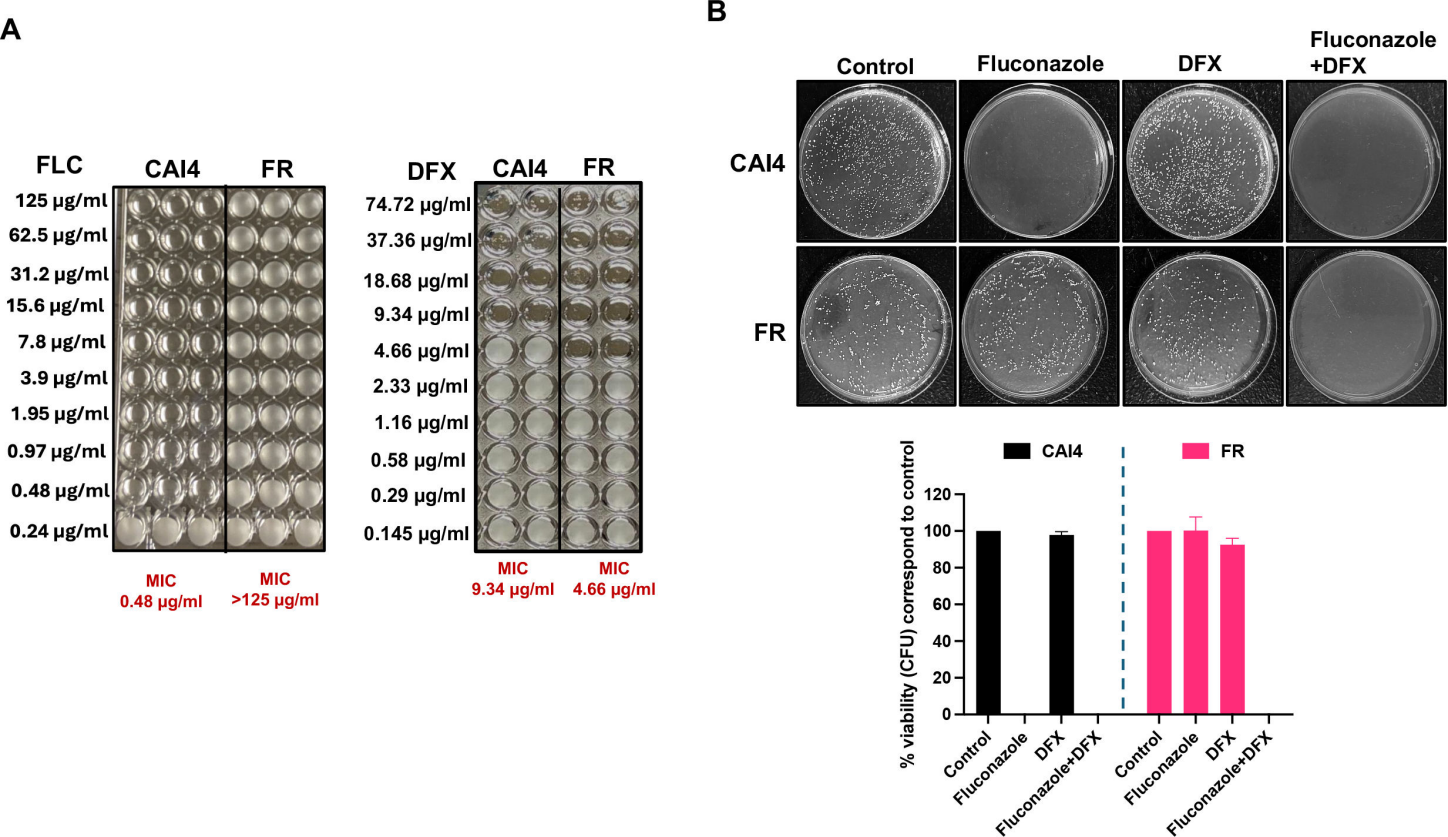
FR strain is more sensitive to iron chelation that can synergize with fluconazole-treatment to overcome resistance. (**A**) *In vitro* susceptibility study to fluconazole (0.24–125 µg/mL) and DFX (0.145–74.72 µg/mL) in CAI4 and FR strains. FR strain showed resistance up to more than 125 µg/mL. (**B**) Pictorial representation of the CFU assay for CAI4 and FR strains under different conditions—control (YNB media only), fluconazole (125 µg/mL), sublethal deferasirox (DFX; 2.33 µg/ml), and fluconazole (125 µg/mL) + sublethal DFX (2.33 µg/mL). Graphical representation showed the percentage viability of CAI4 and FR strains relative to their control. The results of four independent biological repeats are represented as means ± SEM.

Fluconazole resistance of our FR strain was confirmed by determining the minimum inhibitory concentration (MIC) of fluconazole against it as well as its parent strain CAI4, over a range of concentrations (125–0.24 µg/mL). CAI4 strain was susceptible to fluconazole, with an MIC of 0.48 µg/mL, while the FR strain showed significant resistance to fluconazole, with an MIC >125 µg/mL (Fig. 1A, left). Colony forming unit (CFU) viability assay on agar plates with or without 125 µg/mL fluconazole further confirmed the fluconazole sensitivity pattern of FR and CAI4 strains (Fig. 1B).

In contrast, the FR strain was more sensitive to DFX, compared to CAI4, as the DFX MIC for the FR strain was twofold lower (4.66 µg/mL) than CAI4 DFX MIC (9.34 µg/mL) (Fig. 1A, right). At a sublethal dose (2.33 µg/mL) of DFX, which is lower than the MIC for both strains, the FR and CAI4 strains showed robust growth in CFU viability assay. However, 2.33 µg/mL of DFX was able to effectively synergize with 125 µg/mL fluconazole to completely inhibit the growth of FR strain (Fig. 1B). Thus, DFX alone or in combination with fluconazole has the potential to kill FR *C. albicans*.

### FR *C. albicans* has enhanced intracellular iron levels

**Fig 2:**
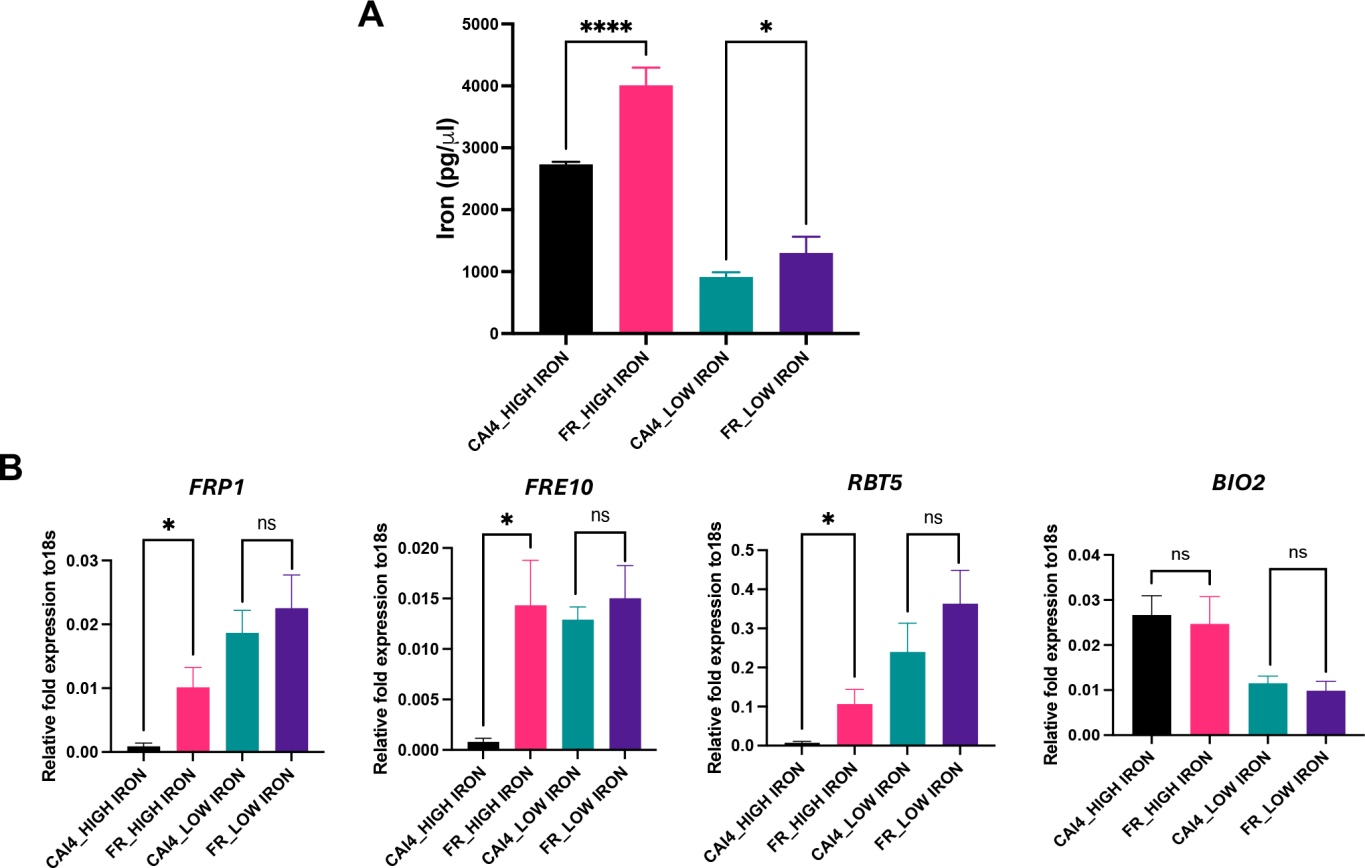
FR strain shows high intracellular iron levels supported by induction of iron-uptake genes. (**A**) CAI4 and FR strains were exposed to high-iron and low-iron conditions and intracellular iron level was measured by calorimetric assay. Results of three biological repeats with triplicates are represented as mean ± SEM. Statistical significance analysis was assessed by one-way ANOVA, *P  <  0.05, ****P < 0.0001. (**B**) Expression study of iron uptake and iron utilization related genes in CAI4 and FR strains by real-time PCR analysis. 18s rRNA gene was used to measure the relative fold expression of genes. The results of four independent biological repeats with triplicates are represented as means ± SEM. Statistical significance analysis was assessed by one-way ANOVA.

Greater sensitivity of FR strain to iron chelation by DFX (Fig. 1) suggested a need for higher intracellular iron by this strain. To assess if that is the case indeed, we measured intracellular iron levels in FR and CAI4 strains, under low and high iron. FR strain showed significantly higher iron levels, compared to CAI4, under both iron conditions (Fig. 2A). However, the statistical significance in fold difference was much greater under high iron than under low iron (*P* < 0.0001 and *P* < 0.05, respectively). To understand how FR strain accumulates significantly higher iron levels, gene expression analysis of iron uptake genes (*FRP1*, *FRE10*, and *RBT5*) was performed. The pattern of expression of iron acquisition genes followed the intracellular iron levels (Fig. 2A), with greater expression in the FR strain, compared to CAI4, especially under high iron (Fig. 2B). Expression of an iron utilization gene, *BIO2*, did not show any difference between the two strains. This shows that the FR strain actively accumulates more iron, without changes in its usual iron utilization processes, suggesting an increased demand for specific purposes.

### Accumulated intracellular iron enhances membrane sterols and cell wall β-glucan levels and exposure in FR *C. albicans*

**Fig 3:**
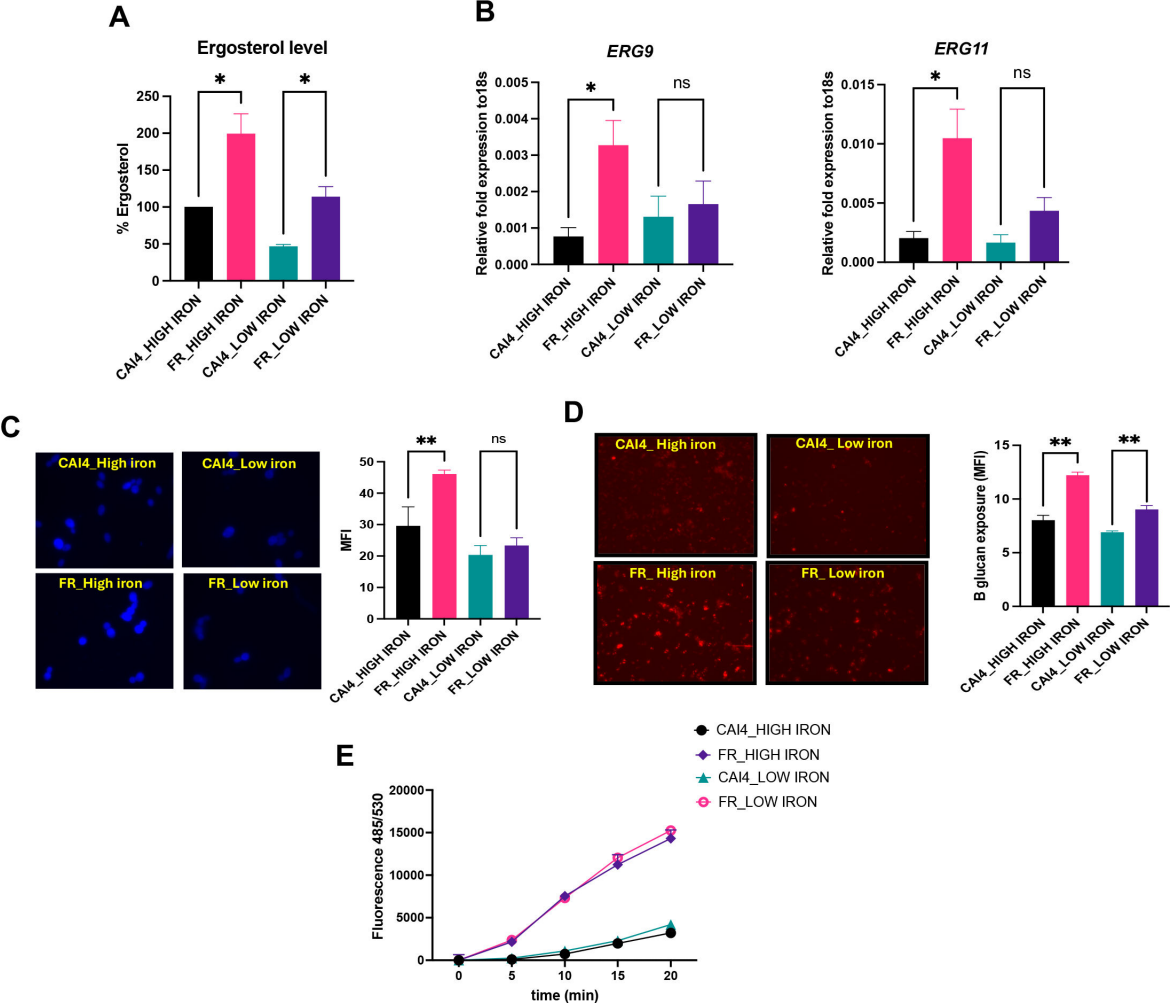
Iron enhances ergosterol synthesis as well as influences β-glucan architecture in FR strain. (**A**) Percent ergosterol levels of CAI4 and FR strains were measured in high and low iron. The results of two independent biological repeats are represented as means ± SEM. (**B**) Gene expression study of ergosterol biosynthesis pathway-related genes was assessed in CAI4 and FR strains under high and low iron by real-time PCR analysis. 18s rRNA gene was used to measure expression of genes. The results of four independent biological repeats with triplicates are represented as means ± SEM. (**C**) β-glucan level and (D) β-glucan exposure were measured by aniline blue dye and anti-β-1,3-glucan antibody in high iron and low iron, respectively. Mean fluorescence intensities (MFIs) for *n* > 150 cells from three independent biological experiments are represented as mean ± SEM. (**E**) Efflux study in CAI4 and FR strains was performed in high and low iron conditions. 2% glucose was added after 30 min of R6G incubation to a glucose-free PBS. The experiment was repeated two times. The graphs show the results of one representative experiment. Statistical significance analysis was assessed by one-way ANOVA, *P < 0.05, **P < 0.01.

Next, we interrogated whether the FR strain is enhancing intracellular iron specifically to induce iron-mediated upregulation of membrane sterols and cell wall glucans that can help mitigate the effects of fluconazole. Thus, we measured the levels of membrane ergosterol and expression of key ergosterol biosynthesis genes (*ERG11* and ERG9) as well as the levels and exposure of β-glucan, in FR and CAI4 strains. Membrane ergosterol levels and *ERG9* and *ERG11* expression were higher in the FR strain, compared to CAI4 (Fig. 3A and B), under both iron conditions. FR strain also showed significantly enhanced β-glucan levels, under high iron (Fig. 3C) and exposure under both iron conditions (Fig. 3D), compared to CAI4. This suggests that the high iron accumulation in the FR strain induced enhanced ergosterol biosynthesis as well as glucan exposure.

We also measured efflux pump responses in CAI4 and FR strains, under high and low iron conditions, using rhodamine 6G dye. As expected, efflux levels were significantly higher (4.46-fold under high iron and 3.62-fold under low iron) in the FR strain, compared to CAI4 (Fig. 3E). However, this difference was independent of high and low iron conditions in both strains. Thus, the classical efflux pump-mediated mechanism of fluconazole resistance is iron-independent, while resistance mechanisms related to changes in membrane and wall components are iron-dependent.

### Fluconazole resistance in *C. albicans* is reduced in a low-iron host

**Fig 4:**
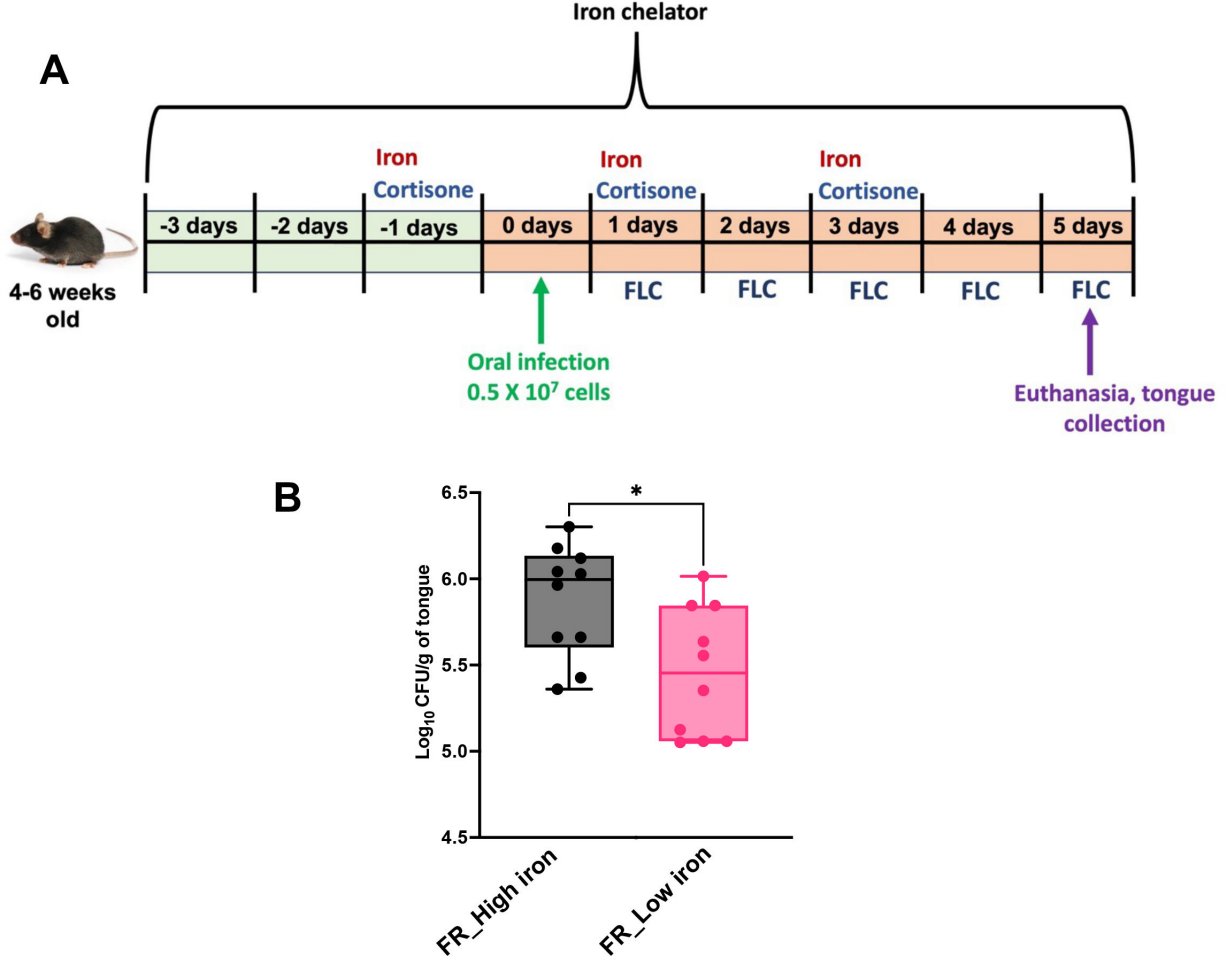
Low-iron mice reduce FR strain fungal burden in murine OPC model. (**A**) Pictorial representation of high-iron and low-iron murine OPC model infected with FR strain followed by treatment with fluconazole (FLC) 1 mg/kg/day. (**B**) CFU/gram of tongue tissue was obtained from *C. albicans*-infected mice. The data were pooled from mice (*n* = 10) and represented as mean ± SEM by Box-and-Whisker plot. Statistical analysis was performed by Mann-Whitney test, *P < 0.05.

Using our murine oropharyngeal candidiasis (OPC) model (Fig. 4A), we next assessed fluconazole resistance as a function of host iron levels. Mice with high iron (achieved with iron-dextran treatment) or low iron (achieved with DFX treatment) were infected with the FR strain and treated with fluconazole. Fungal burden in murine tongue (log_10_ CFU/g of tongue tissue) was significantly higher in high-iron mice (log_10_ 5.873), compared to low-iron mice (log_10_ 5.454); *P* value of 0.0107, Mann-Whitney test (Fig. 4B) after fluconazole treatment. This suggests that maintaining fluconazole resistance requires high environmental iron and a reduction in that iron supply negatively impacts fluconazole resistance.

### DFX synergizes with fluconazole to kill FR clinical strains of *C. albicans*

**Fig 5:**
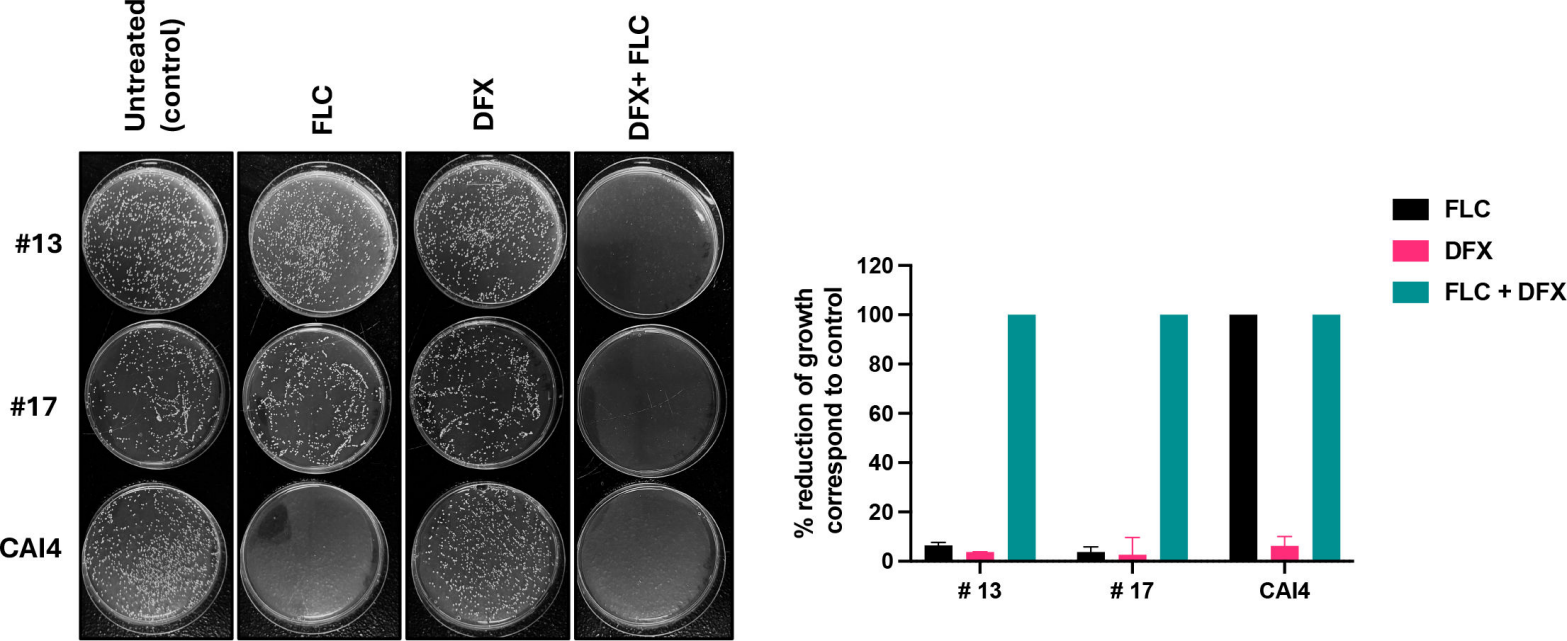
Synergistic killing of FR clinical strains of *C. albicans* by fluconazole and DFX. (Left) Synergistic effect of fluconazole and iron chelator DFX was studied in *C. albicans* FR clinical strains and CAI4 by CFU study. Control (YNB media only), fluconazole (125 µg/mL), sublethal deferasirox (DFX; 2.33 µg/mL), and fluconazole (125 µg/mL) + sublethal DFX (2.33 µg/mL) were used. (Right) Percentage reduction of growth was measured relative to their control CFU. The results of two independent biological repeats are represented as means ± SEM.

We also assessed the efficacy of DFX alone or in synergy with fluconazole against FR clinical isolates of *C. albicans*. FR clinical strains were exposed to either 2.33 µg/mL of DFX alone or in combination with 125 µg/mL fluconazole, followed by CFU quantification. Results showed that the FR clinical strains were able to survive the DFX concentration (Fig. 5) that was also sublethal to our lab-generated FR and CAI4 strains (Fig. 1). However, DFX synergized with fluconazole to kill these strains, while fluconazole alone was unable to eradicate them (Fig. 5), similar to what was observed for our FR strain (Fig. 1). This suggests DFX can synergize with fluconazole to inhibit the growth of FR clinical strains.

## DISCUSSION

The role of iron availability in azole resistance has been observed in various previous studies. Susceptibility of *C. albicans* to fluconazole increased in the presence of various iron chelators, such as bathophenanthrolinedisulfonic acid, deferoxamine, ferrozine, or DIBI (23, 26, 28), while desferrioxamine was also shown to synergistically enhance the susceptibility of *C. glabrata* to fluconazole (29). Our data extend these findings to the most commonly and currently used FDA-approved iron chelator on the market by showing that DFX worked alone or in synergy with fluconazole to kill FR *C. albicans* strains (Fig. 1 and 5). This underscores the significance of iron chelation as an adjunctive therapy in the treatment of FR fungi.

*C. albicans* reprioritizes metal handling during fluconazole stress, including that for iron (30). In this study, we revealed that FR strain of *C. albicans* actively accumulates iron, supported by an increase in the expression of iron uptake genes (Fig. 2) and interfering with that accumulation by using an iron chelator completely abrogated its fluconazole resistance (Fig. 1B). Similar phenomenon has been observed in another pathogenic yeast, *Cryptococcus neoformans*, where a strain with impaired iron uptake was shown to be more vulnerable to azoles (27).

A previous study showed that the increased intracellular iron in fluconazole-treated *C. albicans* cells was not retained in labile pools and instead was incorporated into structures not observable by electron paramagnetic resonance (30). Here, we showed that while iron uptake genes were indeed upregulated, leading to increased intracellular iron (Fig. 2A and B), the iron utilization gene did not show any change (Fig. 2B). In addition, we observed no significant differences in the growth rate of the FR strain under high and low iron conditions (data not shown). In line with the previous findings (30), this suggests that elevated iron levels are redistributed for the production of very specific iron-related proteins. Increased expression of ergosterol biosynthesis genes (Fig. 3A) that is needed for fluconazole resistance suggests that lanosterol 14α-demethylase (Cyp51), an iron-requiring heme protein (31) involved in ergosterol biosynthesis, is upregulated (32). Thus, the increased iron demand in a fluconazole resistance strain might help overcome resistance by greater synthesis of heme-containing ergosterol biosynthesis machinery.

Mitochondria is an important site for Fe-S cluster and heme biosynthesis (33) and iron import into the mitochondria has been shown to positively impact fluconazole resistance (33–35). However, increased iron import into the mitochondria raises the concern for iron-induced mitochondrial reactive oxygen species (ROS) (36). We have recently shown a specific role for alternative oxidase (AOX) in scavenging high iron-mediated mitochondrial ROS (37). Interestingly, AOX has also been shown to play a role in fluconazole resistance, since blocking the alternative respiratory pathway (through inhibition or deletion of AOX) induced susceptibility to antifungal azoles (38).

Besides membrane sterols, cell wall glucans also affect fluconazole susceptibility, and glucan synthesis has been associated with *C. albicans* biofilm resistance to azoles (16). Higher levels of β-glucans absorb fluconazole and inhibit its penetration to the site of action (12). We previously showed that high iron signals increase β-glucan levels and exposure (24), and our present study shows the accumulation of iron in the FR strain had the same effect (Fig. 3B). This underscores that iron-mediated changes in β-glucan can also affect fluconazole resistance in the FR strain.

Overall, our results suggest that active iron accumulation may serve dual purposes in maintaining fluconazole resistance: supporting heme-related ergosterol synthesis machinery and increasing glucan levels to prevent fluconazole entry and retention inside the cell. In addition, the fact that our FR strain was more sensitive to iron chelation alone, compared to its parent strain (Fig. 1), suggests that due to continuous pressure to maintain high cellular iron, FR strains may evolve to become overly sensitive to iron chelation itself. The mechanism of this evolution to be iron chelation sensitive requires further investigation and can offer novel insights into the link between fungal drug resistance and iron metabolism. Regardless, the present study offers a therapeutic strategy for tackling the growing issue of FR *C. albicans* infections. This study highlights the active induction of iron uptake as a mechanism to promote iron-mediated processes that sustain fluconazole resistance and the role of iron chelator DFX in combating fluconazole-resistance.

## MATERIALS AND METHODS

### Fungal strains, culture conditions, and animals

*C. albicans* CAI4 + URA strain (*ura3::imm434/URA3*) was used as the parent strain. The FR strain was generated by passaging in our laboratory, as described here: 1 × 10^6^ cells/mL of CAI4 + URA cells were inoculated overnight in liquid yeast nitrogen base (YNB, 427-522; MP Biomedicals) medium and were streaked the next day on YNB plates with 128 µg/mL fluconazole. After 48 h, a single colony was restreaked on YNB plates with 128 µg/mL fluconazole. After 48 h, a single colony from this round was restreaked on YNB plates with 256 µg/mL fluconazole. An isolated colony from this plate was then used to inoculate an overnight liquid culture to make stocks the following day. This strain was designated as the FR strain used in this study and maintained in the presence of 128 µg/mL fluconazole. Deidentified clinical FR strains (39) were received from Dr. Damian Krysan.

For low and high iron experiments, *C. albicans* exponential phase cells were prepared in yeast nitrogen base (YNB; 4027-112; MP Biomedicals) limited iron medium with 2% glucose as low iron medium or the same with the addition of 100 µM FeCl_3_·6H_2_O as high iron medium. An experiment involving animals utilized 4- to 6-week-old C57BL/6 female mice sourced from Jackson Labs, Bar Harbor, ME, USA. Animals were treated humanely in accordance with the protocols approved by the Institutional Animal Care and Use Committee (IACUC protocol 5079) of Temple University.

### Antifungal susceptibility assay

For evaluation of fluconazole drug sensitivity toward *C. albicans*, CAI4 and FR strains were grown exponentially and diluted to 1 × 10^4^ cells/mL in YNB and incubated at 30 ^ᵒ^C for 48 h in the presence of fluconazole. About 0.24–125 µg/mL concentrations of fluconazole were used in 96-well plates for MIC determination. The MICs were determined as the lowest concentration that inhibited ≥90% of the *C. albicans* cells.

For CFU study, FR and CAI4 strains were grown overnight in YNB medium with or without fluconazole respectively. The next day, 50 µL of culture (from 1 × 10^4^ cell suspension) was plated on LIM-agar plates containing 128 µg/mL fluconazole alone or in combination with 2.33 µg/mL DFX and further incubated at 30°C for 48 h for CFU determination.

### Intracellular iron estimation

*C. albicans* cells were cultured in metal-free tubes (3194-335-001-9, Labcon) overnight in high and low iron conditions. The next day, the cells were diluted to 0.3 OD and incubated in their respective iron medium for 4 h at 30°C and logarithmic growth phase cultures were pelleted, washed three times, and normalized to OD_600_ to obtain an equal number of cells. Further, cell lysates were prepared by bead beating with glass beads, using Fast Prep-24 (MP Biomedicals). The cell debris was removed by centrifugation at 13,000 RPM for 5 min. The amount of protein in cell lysates was measured using the Pierce BCA (Bicinchoninic acid) Protein Assay (23227, Thermo Scientific). After normalizing protein levels, an equal amount of cell lysates was used for total intracellular iron (Fe^2+^ and Fe^3+^) quantification by an iron assay kit (MAK025, Sigma-Aldrich), according to the manufacturer’s protocol.

### Real-time PCR

RNA was extracted from logarithmic grown *C. albicans* cells cultivated under high and low iron conditions, using a Qiagen RNA isolation kit (74134, Qiagen) via bead-beating in lysis buffer (37). Briefly, 10 mL culture pellet of *C. albicans* cells was subjected to bead-beating (six to seven cycles, 6 m/s) with 0.45-mm-diameter glass beads using a FastPrep-24 instrument (MP Biomedicals). Total RNA was isolated using a Qiagen RNeasy mini kit, and genomic DNA contamination was eliminated as per the manufacturer’s protocol. Total RNA quantification was performed using a NanoDrop machine. About 1 μg of DNA-free RNA samples was used for complementary DNA (cDNA) synthesis with the iScriptTM cDNA synthesis kit (1708891, Biorad). Further, an equal volume (1 µL) of generated cDNA was used for determining transcript levels via reverse transcription real-time PCR (RT-PCR) using gene-specific primers and SYBR Green PCR Supermix (1725124, Bio-Rad). Relative mRNA quantities for the target genes and the housekeeping gene (18s) were determined by Applied Biosystem, Thermo Quant Studio 5. Gene expression levels of genes of interest were normalized to the level of 18S rRNA gene in the same sample for each respective condition, and the results were reported as the mean of triplicate samples ± SEM.

### Ergosterol quantification

Briefly, CAI4 and FR strains were cultured under low and high iron conditions for 4 h at 30°C with shaking. After incubation, the cells were harvested by centrifugation at 4,000 rpm for 5 min at 4°C, washed with phosphate-buffered saline (PBS), and transferred to pre-weighed tubes to determine the net wet weight of the cell pellet. For lipid extraction, 1.5 mL of 25% potassium hydroxide in ethanol solution was added to the cell pellets, followed by incubation at 85°C for 1 h in a heat block. The tubes were then cooled to room temperature, and lipids were extracted by adding a mixture of 0.5 mL sterile Milli-Q water and 1.5 mL n-heptane (22583, Sigma-Aldrich). The mixture was vortexed vigorously for 3–5 min, centrifuged, and the supernatant was collected to estimate ergosterol levels. Optical density was measured at 282 nm using a spectrophotometer. The ergosterol content was quantified using a calibration curve prepared with standard ergosterol (117810050, Thermo Scientific) in the range of 3–0.18 mg. Results were expressed as the percentage change in ergosterol levels under high and low iron conditions for the CAI4 and FR strains.

### β-glucan level and exposure measurements

Briefly, 1 × 10^7^ exponential cells of CAI4 and FR strains were grown in low and high iron conditions and fixed with 4% paraformaldehyde for 30 min in PBS. After three washes with PBS, the cells were fixed and stained with 0.1 mg/mL of Aniline Blue (100-1; Bioscience supplies) for β-glucan level estimation, as described previously (24), followed by incubation at room temperature for 30 min. Images were taken with a fluorescence microscope at 100 × magnification and quantified by measuring mean fluorescence intensities (MFIs) using the ImageJ software. Exposed β-1,3-glucan in fixed cells was measured by staining with a primary β-1,3-glucan antibody (Bioscience Supplies, Australia) and a secondary goat anti-mouse antibody conjugated to Cy3 (Jackson Immuno Research), as described previously (22). Images were captured using an EVOS inverted microscope. MFIs for *n* > 150 cells from at least three independent biological experiments are represented as mean ± SEM.

### Assessment of efflux activity

Approximately 0.3 OD yeast cells (CAI and FR strains) were grown overnight in both high and low iron conditions. The next day, the cells were re-cultured in fresh high and low iron media, respectively, and grown for 4 h. The cells were then pelleted, washed two times with PBS (pH 7.0, without glucose), and resuspended to a concentration of 10^8^ cells/ml. These cell suspensions were incubated at 30°C with shaking (220 rpm) for 120 min in glucose-free PBS. The de-energized cells were washed, resuspended in glucose-free PBS, and then rhodamine 6G was added to a final concentration of 10 µM. After incubation at 30°C for 30 min, the cells were washed two times and resuspended in glucose-free PBS to a concentration of 10^8^ cells/mL. Energy-dependent efflux was measured following the addition of glucose to a final concentration of 2%. At 5-min intervals, 1 mL of the cell suspension was removed by centrifugation, and the fluorescence of the supernatant was recorded at an excitation of 485/20 nm and an emission of 528/20 nm. Glucose-free controls were included in all experiments.

### Mice OPC study

For measuring *in vivo* fungal burden under high-iron and low-iron conditions, a previously described immunosuppressed murine OPC model was used (37). Briefly, female C57BL/6 mice aged 4–6 weeks were immunosuppressed by subcutaneous injections of 225 mg/kg of cortisone acetate (C3130; Sigma) on days −1, 1, and 3. In the high-iron group (24), mice were intraperitoneally supplemented with iron-dextran (D8517; Sigma) at 10 mg/kg on days −1, 1, and 3. For the low-iron group, DFX treatment (QA-8243; CombiBlock) was administered via drinking water (0.07 mg/mL) with 2% dextrose from days −3 to 5. Fluconazole was administered at 1 mg/kg/day in both high-iron and low-iron mice groups. On the day of infection (day 0), mice were anesthetized and sublingually infected for 45 min with a 5 × 10^6^ cells/mL suspension of FR. On day 5, mice were euthanized, and tongues were collected, weighed, homogenized in PBS, serially diluted, and plated on YPD agar plates containing streptomycin/penicillin (SV30010; HyClone). These plates were then incubated at 30°C for 48 h. The fungal burden in the tongue tissue was reported as the mean log_10_ values of CFU/g of tongue tissue. The data were pooled from mice (*n* = 10) and represented as mean ± SEM by Box-and-Whisker plot. Statistical analysis was performed by Mann-Whitney test, *P < 0.05.

### Statistical analysis

All the experiments were performed in at least two biological repeats with triplicates, and the results were expressed as mean ± standard error (SEM). Data were analyzed using the Mann-Whitney test or one-way ANOVA, and *P* values lower than 0.05 were considered significant.
